# Comparative Methods to Improve the Detection of *BRAF* V600 Mutations in Highly Pigmented Melanoma Specimens

**DOI:** 10.1371/journal.pone.0158698

**Published:** 2016-07-28

**Authors:** Eric Frouin, Thierry Maudelonde, Romain Senal, Marion Larrieux, Valérie Costes, Sylvain Godreuil, Julie A. Vendrell, Jérôme Solassol

**Affiliations:** 1 CHU Poitiers, Department of Biopathology, Poitiers, France; 2 CHU Montpellier, Arnaud de Villeneuve Hospital, Department of Pathology, Montpellier, France; 3 Institut du Cancer de Montpellier, Department of Biopathology, Montpellier, France; 4 CHU Montpellier, Arnaud de Villeneuve Hospital, Department of Bacteriology, Montpellier, France; 5 IRCM, Institut de Recherche en Cancérologie de Montpellier, Montpellier, France; 6 INSERM, U1194, Montpellier, France; 7 Université de Montpellier, Montpellier, France; Rutgers University, UNITED STATES

## Abstract

Genotyping *BRAF* in melanoma samples is often challenging. The presence of melanin greatly interferes with thermostable DNA polymerases and/or nucleic acids in traditional polymerase chain reaction (PCR)-based methods. In the present work, we evaluated three easy-to-use strategies to improve the detection of pigmented DNA refractory to PCR amplification. These pre-PCR processing methods include the addition of bovine serum albumin (BSA), the dilution of DNA, and the purification of DNA using the NucleoSpin^®^ gDNA Clean-up XS Kit. We found that *BRAF* genotyping in weakly and moderately pigmented samples was more efficient when the sample was processed with BSA or purified with a NucleoSpin^®^ gDNA Clean-up XS Kit prior to PCR amplification. In addition, the combination of both methods resulted in successful detection of *BRAF* mutation in pigmented specimens, including highly pigmented samples, thereby increasing the chance of patients being elicited for anti-BRAF treatment. These solutions to overcome melanin-induced PCR inhibition are of tremendous value and provide a simple solution for clinical chemistry and routine laboratory medicine.

## Introduction

The need has risen for a reliable test detecting *BRAF* V600E and V600K mutations for treatment with vemurafenib, given that this drug has been approved by the Food and Drug Administration (FDA) and the European Medicines Agency for treatment of advanced metastatic melanoma as an inhibitor of V600 mutants [1].

Usually, *BRAF* genotyping is performed in routine laboratories using a combination of in-house techniques based on polymerase chain reaction (PCR), such as High Resolution Melting (HRM) or Sanger sequencing. However, assuming adequate amounts of DNA are present, failure to achieve a sequencing result occurs in 7–10% of cases [2–4]. This issue is partially due to the presence of DNA inhibitors that are not eliminated during the DNA purification step. For example, melanin can inhibit PCR results even at very small amounts [5]. Many efforts have been devoted to the development of pre-treatment procedures to generate PCR-compatible samples. These procedures include standard organic extractions, cesium chloride purification, urea purification, and CTAB extraction [6]. However, these methods are usually complicated, time-consuming, and require experience. In addition, they are often too difficult to implement for the large number of samples routinely performed in clinical laboratories. In addition, they have not been specifically developed against the inhibitory activity of melanin. Some proposed, easy-to-use alternatives include immunocapture/chromatographic column purification, amplification facilitators such as BSA, and DNA dilution [7–10]. However, the assessment of these “pre-cleaning” methods in melanin-pigmented samples has been poorly investigated to date, and precise procedures vary greatly between hospital routine laboratories.

Considering these factors, the aim of the present study was to evaluate easily applicable pre-PCR or PCR methods able to overcome *BRAF* amplification failures in routine, thereby enhancing detection of *BRAF* V600 mutations in melanin-pigmented melanoma samples that are usually refractory to PCR amplification and genotyping.

## Materials and Methods

### Cell culture

The human melanin-free 1676 melanoma cell line and the *BRAF*-wildtype LNCaP and the *BRAF*-mutated WiDR cell lines were obtained from the American Type Culture Collection (Manassas, USA) and cultured as recommended.

### Tissue sample collection

Fifty-nine formalin-fixed paraffin-embedded (FFPE) melanoma tissue samples were submitted to the University Hospitals of Montpellier (France) and of Poitiers (France) for *BRAF* mutation analysis. Patients were obtained from the department of Biopathology in protocols approved by the institutional review board of the University Hospital of Montpellier. For this non-interventional study, a verbal informed consent statement from all individuals prior to their participation in the study in agreement with the University Hospital of Montpellier ethical review committee was obtained. Only verbal consent is relied on the French bioethics decree N° 2007–1220 published in the official journal of the French Republic. Authors do not have access to or collect any identifying information related to these samples. Results of the supplemental experiments for *BRAF* mutation determination were collected blindly, anonymously, without any feedback to attending physicians while molecular analyses had been previously performed as standard testing for patients' management and diagnosis.

Microscopic evaluation confirmed these samples had high melanin content within the tumor and stromal cells. All lesions were processed in the Departments of Pathology using standard techniques. For each sample, the tumor cell percentage, necrosis, and melanin content were recorded by one pathologist (EF) using hematoxylin and eosin stains (H&E) typically used in routine diagnosis. Two independent sets of samples were analyzed. The specimen characteristics are shown in Table 1.

**Table 1:** Patient and specimen characteristics.

Characteristics	Set 1 (n = 9)		Set 2 (n = 50)	
	n	%	n	%
Sex				
Male	7	78	29	58
Female	3	33	21	42
Average age (years)	65.4		63.2	
Melanoma type				
Primary	3	33	10	20
Metastatic	6	67	40	80
Brain	2	22	15	30
Skin	2	22	13	26
Node	1	11	4	8
Other	1	12	8	16
Tumor cell content				
<80%	4	44	12	24
≥80%	5	56	38	76
Microscopic melanin rate				
Sample + (5 to 15%)	3	33	10	20
Sample ++ (20 to 45%)	3	33	22	44
Sample +++ (>50%)	3	33	18	36
Necrosis rate				
Sample 0	8	89	42	84
Sample + (<10%)	1	11	5	10
Sample ++ to +++ (>10%)	0	0	3	6

### Genomic DNA (gDNA) extraction and pre-PCR treatment protocols

For the samples generated from cultured cell lines, gDNA was extracted using the QIAamp DNA Mini Kit (Qiagen, Hilden, Germany) following the manufacturer’s recommendations. Chemically synthesized melanin from Sigma (Courtaboeuf, France) was dissolved in distilled water to a final concentration of 2 mg/ml, vortexed extensively, sonicated in a water bath at room temperature for 10 min, and used at different concentrations to mimic melanin contamination that occurs in tissue samples. For tumor samples, tissue punches were obtained from paraffin blocks using a 1 mm needle, and gDNA extraction was performed using the QIAamp DNA FFPE Tissue Kit (Qiagen). The extracted DNA was quantified using a NanoDrop spectrophotometer (Thermo Scientific, Wilmington, NC, USA).

To circumvent PCR inhibition, bovine serum albumin (BSA) from New England BioLabs (Hitchin, UK) and the NucleoSpin^®^ gDNA Clean-up XS Kit (Macherey-Nagel, Betheleham, PA, USA) were used. For tumor samples, BSA was added to the PCR reactions at a final concentration of 0.1 μg/μl.

### *BRAF* amplification in cell line

Oligonucleotide primers were used to determine the status of mutations in *BRAF* exon 15 (forward and reverse primers were 5′-CCTCAGATATAT TTCTTCATG-3′ and 5′-GATCCAGACAACTGTTCAA-3′, respectively). Conventional PCR amplification was performed in a volume of 50 μl containing 1× PCR buffer, 10 μM deoxyribonucleoside triphosphate (dNTP), 10 μM each of the forward and reverse primers, 1 unit of Platinum Taq DNA polymerase (Life Technologies, Darmstadt, Germany), and 20 ng of genomic DNA. The following cycling conditions were used: 95°C for 10 min, 40 cycles of 95°C for 40 s, 60°C for 40 s and 72°C for 1 min, and a final extension at 72°C for 5 min.

### High-resolution melting (HRM) analysis

PCR amplification and HRM analysis were performed on a Rotor-Gene 6000 (Corbett Research, Mortlake, Australia) by using the LightCycler 480 HRM Master Mix Kit (Roche Diagnostics, Meylan, France). The primer sequences designed to amplify the *BRAF* exon 15 region containing BRAF V600 mutations were as follows: forward 5’-TTCATGAAGACCTCACAGTAAAAA-3’ and reverse 5’-bio-CCACAAAATGGATCCA GACA-3’. PCR amplifications were performed in a final volume of 20 μL, including 20 ng of purified gDNA, 10 μL of 2× PCR mix, 3 mM MgCl2 and 200 nM each of the forward and reverse primers. The cycling conditions were as follows: 95°C for 5 min, followed by 50 cycles of 95°C for 15 s, 63°C for 15 s with an initial touchdown program of 0.5°C/cycle during 11 cycles and 72°C for 25 s. The melting conditions included one cycle at 95°C for 1 min, one cycle at 40°C for 1 min, and one cycle at 65°C for 20 s, followed by a gradual increase from 65°C to 95°C at 0.1°C per second. The HRM data were analyzed using the Rotor-Gene 6000 version 1.7 (Corbett Research). The HRM analyses were performed in duplicate for each sample. The normalized melting curves were evaluated, and the samples were compared with the wildtype and mutant controls in a deduced difference plot (S1A Fig). Significant deviations from the horizontal line relative to the spread of the wildtype control were indicative of sequence changes within the analyzed amplicon (S1B Fig). The results were given as the percentage of homology with the LNCaP *BRAF*-wildtype cell line. In our experience, a homology percentage of <70% indicated that a mutation was present in the DNA sample. A homology percentage between 70% and 90% was inconclusive.

### Pyrosequencing assay

Genotyping was also carried out on the pyrosequencing platform PyroMark^™^ Q96 ID instrument (Qiagen) using the Therascreen BRAF Pyro Kit (Qiagen) according to the manufacturer’s instructions. Briefly, a targeted region was PCR-amplified and sequenced. The sequences surrounding codon 600 of the *BRAF* gene served as normalization and reference peaks for quantification and quality assessment of the analysis. The results are given as percentage of mutated amplicons. A percentage of mutation >5% indicated the presence of a mutation in the DNA sample.

### Sanger sequencing

PCR amplifications were performed in a final volume of 25 μL, including 20 ng of purified gDNA, 2.5 μL of 10× PCR buffer, 2 mM of MgCl2 and 200 μM of dNTP, 1.5 U of Platinum Taq DNA polymerase (Life Technologies) and 400 nM each of the forward and reverse primers. The primer sequences were as follows: forward 5’-TGTTTTCCTTTACTTACTACACCTCA-3’ and reverse 5’-CCACAAAATGGATCCAGACA-3’. The cycling conditions used were as follows: 95°C for 10 min, 35 cycles of 94°C for 30 s, 60°C for 30 s and 72°C for 30 s and a final extension at 72°C for 10 min. The products obtained were then treated with ExoSAP-IT (Affymetrix, Santa Clara, CA, USA) according to the manufacturer’s recommendations. The sequencing reactions were performed in a final volume of 10 μl as follows: 2 μl of diluted PCR product, BigDye Terminator v1.1 Sequencing reagents (Life Technologies), and 150 nM each of the forward and reverse primers. The cycling conditions were as follows: 96°C for 2 min, followed by 25 cycles of 96°C for 10 s, 50°C for 5 s, and 60°C for 2 min. Sequencing products were purified using the BigDye Xterminator Purification Kit (Life Technologies) as recommended. Products were then sequenced on a 3130 Genetic Analyzer (Applied Biosystems) and analyzed with the sequencing analysis software (Applied Biosystems).

## Results

### Assessment of *in vitro* procedures to circumvent PCR inhibition

**Fig 1:**
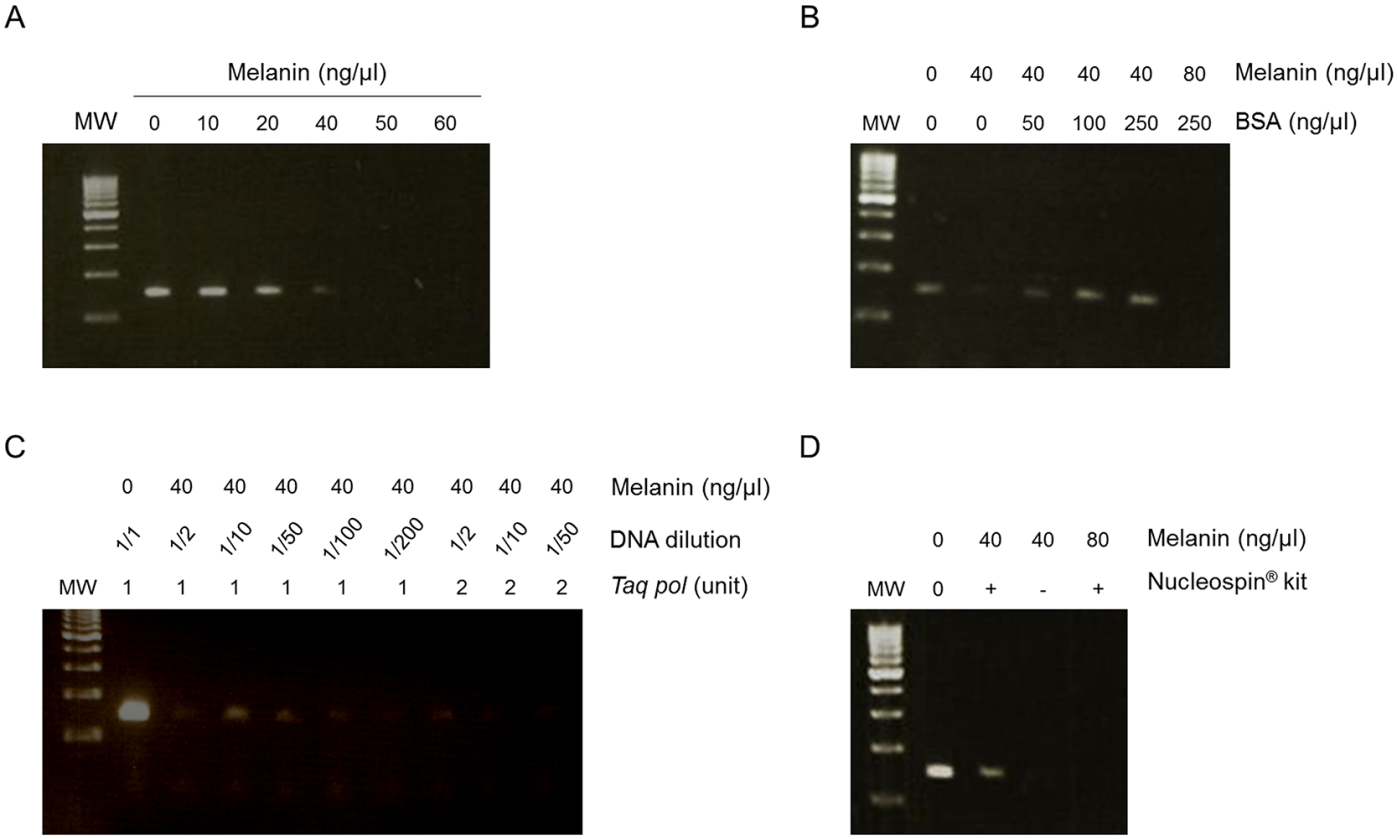
Removal of the inhibitory effects of melanin on PCR amplification. (A) Increasing concentrations of synthetic melanin were added to DNA extracted from 1676 melanoma cell lines. (B) Increasing concentrations of BSA (ng/μl) were added to DNA extracted from cultured cells containing 40 or 80 ng/μl of melanin. (C) The effect of diluting DNA assessed in the presence of 40 ng/μl of melanin and either 1U or 2U of *Taq* polymerase. (D) NucleoSpin^®^ gDNA Clean-up XS Kit used on DNA extracted from cultured cells containing 40 or 80 ng/μl of melanin. PCR amplification of the DNA was monitored on 2% gel agarose electrophoresis with ethidium bromide staining. MW, molecular weight markers.

We first determined the amount of melanin required to inhibit *BRAF* amplification in the 1676 melanoma cell line. As expected, *BRAF* amplification was suppressed when reaction mixtures contained a concentration of 40 ng/μl of melanin, even after 40 cycles of PCR (Fig 1A). When 50 or 100 ng/μl of BSA was added to the reaction mixture, PCR inhibition was only partially inhibited in presence of 40 ng/μl of melanin, whereas 250 ng/μl of BSA totally reversed inhibition (Fig 1B). However, the same BSA concentration could not overcome *BRAF* amplification inhibition when higher concentrations of melanin were included in the reaction, demonstrating that the amount of melanin influences the efficiency of BSA to reverse melanin inhibition *in vitro*. Subsequently, we tested the ability of DNA dilution to lower the PCR-inhibiting effects of melanin. *BRAF* detection was successful in samples containing a total of 20 ng of template DNA using up to 1/10 dilution (Fig 1C). In contrast, higher dilutions of 1/50 resulted in sub-optimal quantities of template DNA that hampered PCR amplification. Increasing *Taq* concentrations slightly facilitated amplification in melanoma extracts, but this value dropped for lower copy templates. Altogether, these data demonstrated that such processes remain hazardous for the management of melanoma samples in routine settings. Optimizations are needed to standardize efficient pre-treatment procedures in accordance with the level of melanin contamination. Finally, the NucleoSpin^®^ gDNA Clean-up XS Kit provided greater efficiency of *BRAF* amplification but did not completely resolve the difficulties presented by these types of samples, notably when higher concentrations of melanin were added (Fig 1D).

### Comparison of 3 DNA pre-PCR treatments in tissue specimens (set 1)

**Fig 2:**
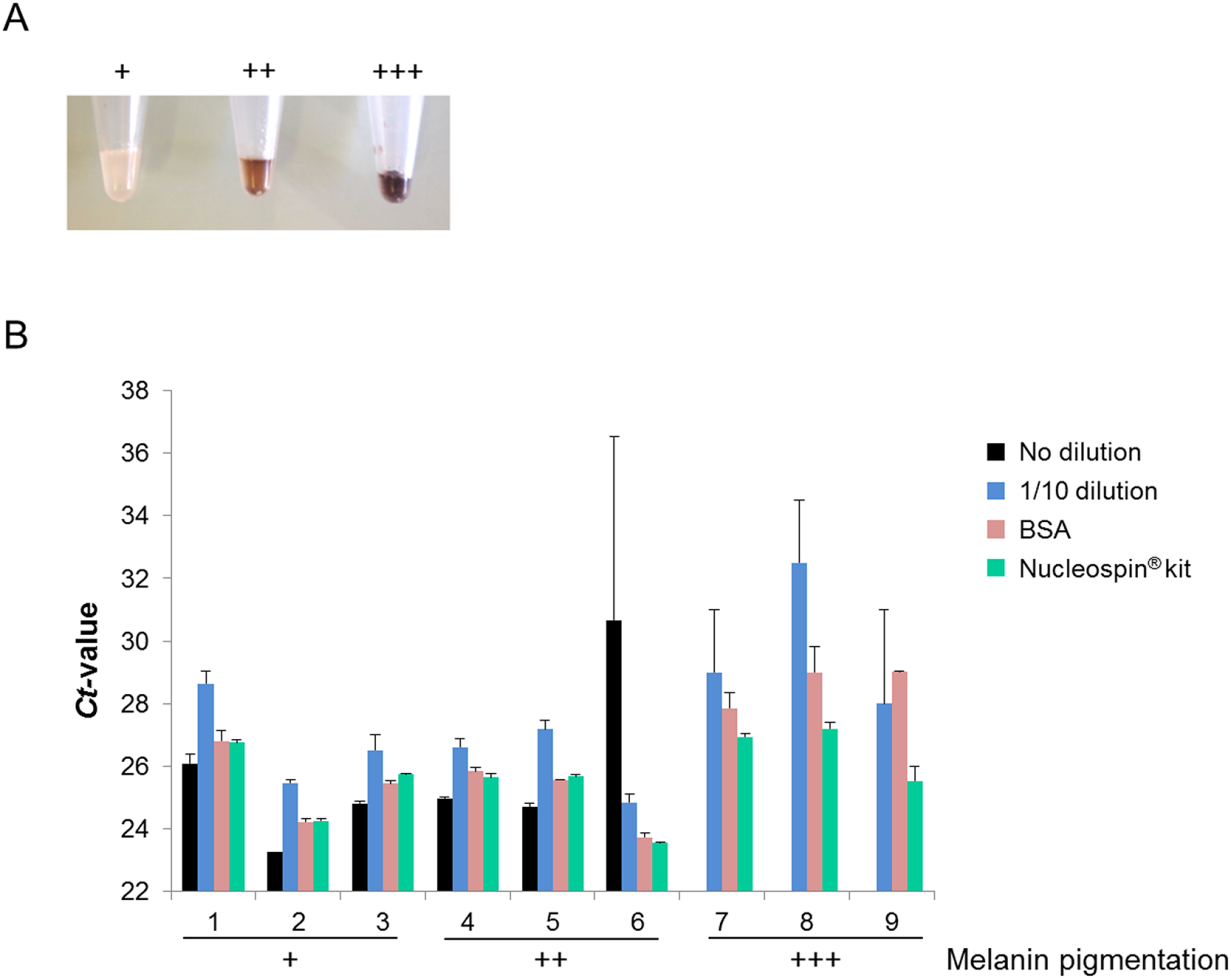
Example of *BRAF* amplification in several tissue samples. (A) Image showing samples with a weak (+), moderate (++), or high (+++) level of melanin contamination after DNA extraction. (B) HRM *C*_*t*_-values for samples harboring different levels of melanin contamination and treated with different pre-PCR procedures.

First, a set of nine tissue samples harboring weak (n = 3), moderate (n = 3) and high (n = 3) melanin levels (Fig 2A) were investigated for *BRAF* mutation detection using the HRM conditions previously determined in cell lines. *Ct*-values are expected to be similar between non-pretreated samples and samples that were pre-treated with BSA and the clean-up Kit, approximately 25 cycles. Moreover, samples that are diluted 1/10 should exhibit a 3.33 cycle difference corresponding to the dilution factor. The results showed that *C*_*t*_-values are directly dependent on the level of melanin pigmentation. Indeed, in weakly pigmented samples, similar *C*_*t*_-values were obtained regardless of the pre-PCR procedure used, including standardized DNA samples that had no pre-treatment processing (Fig 2B). In moderately pigmented samples, *C*_*t*_-values remained concordant between samples processed with the 3 pre-PCR treatments and samples that received no pre-treatment. The only exception was sample 6, which exhibited non-reproducible PCR amplification in absence of treatment. Finally, in highly pigmented samples, *BRAF* amplification systematically failed in all samples processed without pre-treatment. In addition, although *C*_*t*_-values in samples pre-treated with both BSA and the NucleoSpin^®^ Kit remained acceptable, *C*_*t*_-values were discordant and results were less reproducible in diluted samples. Therefore, because this method was not suitable in the presence of high melanin concentrations, the dilution pre-treatment protocol was excluded for the following validation experiments.

### Comparison of BSA and NucleoSpin^®^ Kit procedures for *BRAF* V600 detection in tissue specimens (set 2)

**Fig 3:**
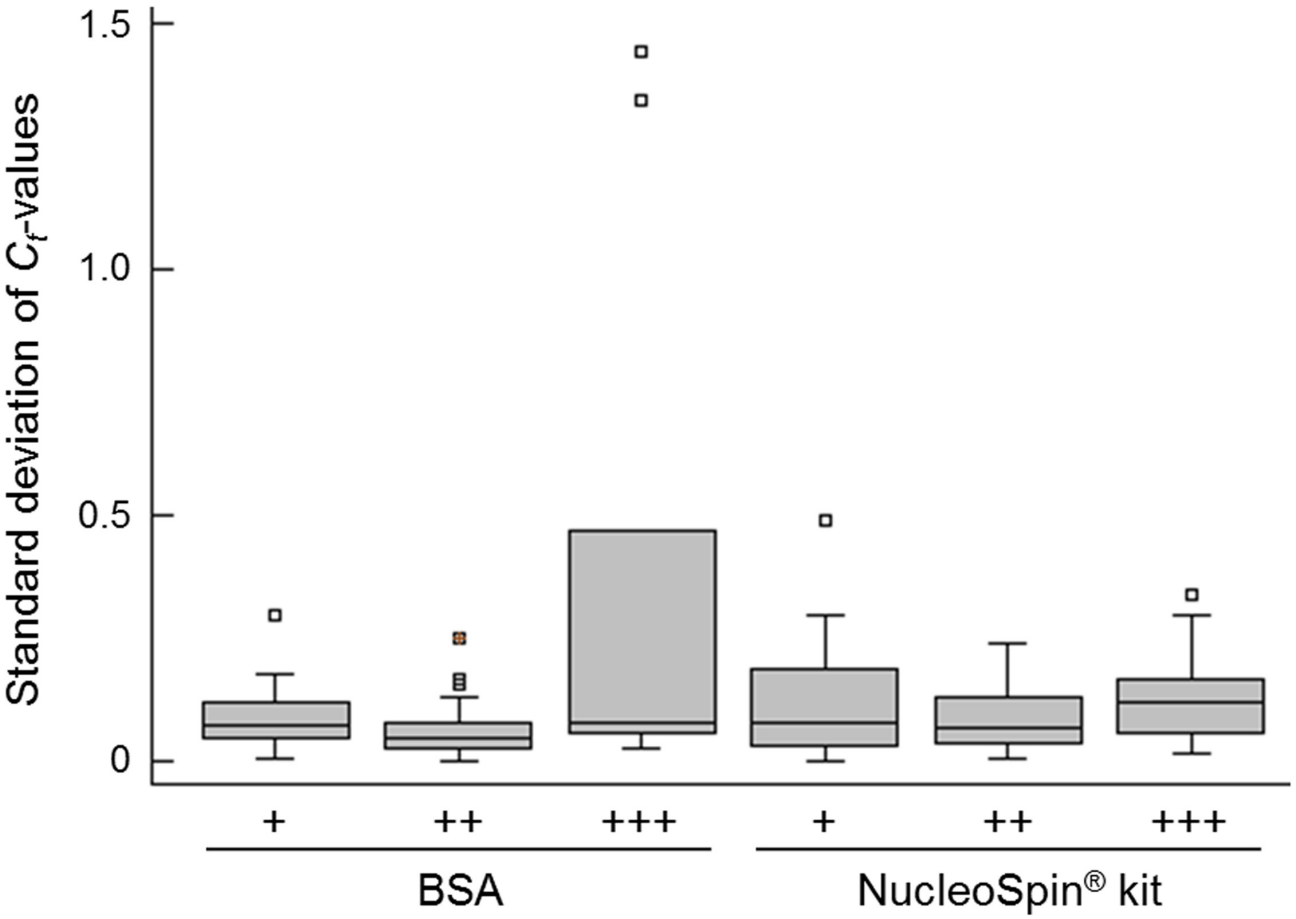
Reproducibility of the HRM *C*_*t*_-values for 50 samples pre-treated with either BSA or the NucleoSpin^®^ Kit. Box-and-whisker plots represent the standard deviation of *C*_*t*_-values obtained for samples with weak (+, n = 20), moderate (++, n = 21), or high (+++, n = 9) melanoma contaminations that were pre-treated with either BSA or the NucleoSpin^®^ Kit. Each sample was analyzed in duplicate.

We used HRM, pyrosequencing, and Sanger sequencing to analyze 50 independent pigmented tissue specimens to determine the presence of *BRAF* V600 mutations. HRM *BRAF* analysis is commonly used in routine laboratories as a screening test to differentiate potentially mutated samples from wildtype specimens. When considering the *C*_*t*_-values obtained for duplicates, we noticed that standard deviations were higher when using BSA pre-treatment when compared to the NucleoSpin^®^ Kit, particularly for highly pigmented samples (Fig 3). However, when we compared the HRM profiles of tissue samples to the *BRAF*-wildtype LNCaP cell line, we observed that HRM results were conclusive in 80% of cases processed with BSA pre-treatment. This was true whatever the degree of pigmentation in the sample eliciting conclusive *BRAF* profiles. In contrast, when using the NucleoSpin^®^ Kit pre-treatment, most of the moderately and highly pigmented DNA samples remained difficult to genotype in half of the cases (Table 2).

**Table 2:** Conclusive results obtained by HRM analysis according to the level of melanin contamination.

Level of melanin contamination	No pre-treatment n (%)	BSA n (%)	NucleoSpin Kit n (%)
**Total (n = 50)**	**24 (48%)**	**40 (80%)**	**24 (48%)**
Weak (n = 20)	13 (65%)	16 (80%)	12 (60%)
Moderate (n = 21)	11 (52%)	16 (76%)	10 (48%)
High (n = 9)	0 (0%)	8 (89%)	2 (22%)

Moreover, when the 50 samples were analyzed with the use of BSA or the NucleoSpin^®^ Kit pre-PCR methods, the correct classification of *BRAF*-mutated or *BRAF*-wildtype occurred in 15 and 6 patients, respectively (Table 3).

**Table 3:** *BRAF* mutation status determined by HRM using DNA treated with BSA or purified using the NucleoSpin^®^ Kit.

	BSA		
	Mutated	Wildtype	Inconclusive^a^
**NucleoSpin**^**®**^ **Kit**			
Mutated	15	0	0
Wildtype	0	6	3
Inconclusive^a^	5	14	7

^a^ Some of the inconclusive HRM results were further analyzed by pyrosequencing

Notably, conclusive HRM results were obtained for samples that were not pre-treated in 13/20 (65%), 11/21 (52%), and 0/9 (0%) of the weakly, moderately, and highly pigmented samples, respectively (Table 2). Although the number of conclusive results seems similar to those obtained using the NucleoSpin^®^ Kit (Table 2), 50% of the inconclusive samples are due to no amplification, totally excluding these samples for all genotyping methods based on PCR (data not shown). This is contrary to the NucleoSpin^®^ Kit, for which all samples were amplified. Moreover, for two of the samples that did not receive pre-treatment, their HRM profiles clearly demonstrated melting curves similar to the mutated cell line, while their pyrosequencing results determined that these samples were *BRAF* wildtype (data not shown).

Next, 17 of the remaining 29 inconclusive samples were analyzed using pyrosequencing. The results showed that this approach could detect *BRAF* mutations in almost all samples, except for two highly pigmented specimens, independently of the DNA pre-treatment procedure used (Table 4). The Sanger sequencing results were inconclusive for 4/17 and 0/17 samples using BSA and the NucleoSpin^®^ Kit pre-treatments, respectively (Table 4). Finally, the two highly pigmented samples that were refractory to pyrosequencing were further analyzed using a combination of the NucleoSpin^®^ Kit followed by BSA treatment. Interestingly, HRM, pyrosequencing, and Sanger sequencing all allowed for the correct genotyping of these samples (Table 5).

**Table 4:** Comparison of BSA and NucleoSpin^®^ Kit procedures for routine detection of *BRAF* mutations.

Sample ID	Level of melanin in DNA samples	BSA						NucleoSpin^®^ kit						Final results^d^
		HRM analysis^a^			Pyrosequencing^b^		Sanger^c^	HRM analysis^a^			Pyrosequencing^b^		Sanger^c^	
		Ct	% of homology with BRAF-WT gDNA	Result	Result	% of mutation	Result	Ct	% of homology with BRAF-WT gDNA	Result	Result	% of mutation	Result	
M1	+	31.41	83.38	Inc.	4%	WT	WT	30.73	90.66	WT	2%	WT	WT	WT
M1	+	31.83	35.58					30.44	95.34					
M2	++	27.15	96.24	WT	2%	WT	WT	26.61	84.62	Inc.	2%	WT	WT	WT
M2	++	27.14	98.61					26.54	89.50					
M3	+++	29.31	84.49	Inc.	3%	WT	Inc.	28.80	80.70	Inc.	2%	WT	WT	WT
M3	+++	29.55	88.77					28.88	90.83					
M4	+++	29.86	96.78	WT	1%	WT	WT	29.55	87.23	Inc.	3%	WT	WT	WT
M4	+++	29.96	96.14					29.35	98.29					
M5	+++	28.67	37.51	Mut	23%	V600E	V600E	28.08	82.75	Inc.	18%	V600E	V600E	V600E
M5	+++	28.65	25.08					27.57	78.19					
M6	+	37.94	90.08	Inc.	2%	WT	WT	35.61	67.08	Inc.	2%	WT	WT	WT
M6	+	No amplification						37.11	97.13					
M7	+++	29.54	92.54	WT	2%	WT	WT	28.56	86.68	Inc.	3%	WT	WT	WT
M7	+++	29.33	90.10					28.88	93.38					
M8	+	28.36	84.79	Inc.	2%	WT	WT	27.64	90.78	WT	1%	WT	WT	WT
M8	+	28.24	99.15					27.46	93.57					
M9	+++	30.45	54.91	Mut	16%	V600E	V600E	30.09	87.03	Inc.	16%	V600E	V600E	V600E
M9	+++	30.54	63.56					30.19	95.37					
M10	+	27.90	96.83	WT	1%	WT	WT	26.83	80.27	Inc.	1%	WT	WT	WT
M10	+	27.77	96.61					27.20	96.88					
M11	+++	33.35	6.71	Mut	23%	V600E	No amplification	28.06	86.52	Inc.	22%	V600E	V600E	V600E
M11	+++	33.34	4.87					28.00	70.88					
M12	+++	26.73	97.15	WT	2%	WT	WT	26.27	74.29	Inc.	2%	WT	WT	WT
M12	+++	26.73	96.60					26.37	83.22					
M13	+	27.74	96.63	WT	2%	WT	WT	28.18	87.80	Inc.	1%	WT	WT	WT
M13	+	27.73	99.45					28.26	82.27					
M14	+	26.87	98.90	WT	1%	WT	WT	26.16	74.00	Inc.	2%	WT	WT	WT
M14	+	26.79	99.13					26.13	79.27					
M15	++	27.74	99.37	WT	2%	WT	WT	27.12	71.23	Inc.	1%	WT	WT	WT
M15	++	27.67	98.38					27.18	74.56					
M16	+++	31.95	82.46	Inc.	13%	V600K	No amplification	29.68	78.1	Inc.	17%	V600K	WT	V600K
M16	+++	32.14	29.86		9%	V600K		29.92	86.28		12%	V600K		
M17	+++	36.41	0.46	Inc.	74%	V600K	No amplification	No amplification		Inc.	N.C.	N.C.	WT	N.C.
M17	+++	38.41	49.25		1%	WT		36.30	83.07		2%	WT		

Inc., inconclusive; WT, *BRAF*-wildtype sample; Mut, *BRAF*-mutated sample; N.D., not done.

^a^ HRM analyses were performed in duplicate. The results were given in percent homology with the LNCaP BRAF-wildtype cell line. Homology > 90%: *BRAF*-wildtype sample; 70% < homology < 90%: inconclusive result; homology < 70%: *BRAF*-mutated sample.

^b^ Pyrosequencing analyses were performed in singlet, except for two samples that were difficult to genotype (M16 and M17).

^c^ Sanger sequencing was also performed in singlet.

^d^ Mutation status resulting from a summary of all the results obtained, regardless of the pre-treatment procedure and the genotyping technic used.

**Table 5:** Combinaison of the Nucleospin^®^ Kit and BSA treatment for routine detection of *BRAF* mutation in highly pigmented samples.

Sample ID	Level of melanin in DNA samples	NucleoSpin^®^ Kit + BSA					
		HRM analysis^a^			Pyrosequencing^b^		Sanger^c^
		Ct	% of homology with BRAF-WT gDNA	Result	% of mutation	Result	
M16	+++	31.57	68.25	Mut	22%	V600K	V600K
M16	+++	31.76	44.80		40%	V600K	
M17	+++	37.52	95.23	WT	1%	WT	WT
M17	+++	37.77	97.79		2%	WT	

^a^ HRM analyses were performed in duplicate. The results were given in percent homology with the LNCaP BRAF-wildtype cell line. Homology > 90%: *BRAF*-wildtype sample; 70% < homology < 90%: inconclusive result; homology < 70%: *BRAF*-mutated sample.

^b^ Pyrosequencing analyses were performed in duplicate.

^c^ Sanger sequencing was performed in singlet.

## Discussion

The sensitivity and accuracy of PCR can be compromised by several factors, including DNA extraction, primers, PCR enzymes, thermocycling conditions, and PCR inhibitors. Each of these parameters can potentially influence PCR amplification of the target amplicons, resulting in different levels of sensitivity in assays used to detect mutations. PCR inhibitors generally exert their effects through direct interaction with DNA or by interfering with thermostable DNA polymerases [11]. Melanin is one of the main inhibitors found in melanoma tissue samples. It is now well established that melanin primarily reduces the ability of thermostable polymerases to extend newly synthesized DNA [12, 13]. This inhibitory effect is conferred by a direct and reversible interaction between the polymerase and melanin [5]. Therefore, detection of *BRAF* mutations can be compromised by the presence of melanin contamination, which can ultimately prevent patients from being classified for vemurafenib treatment.

Consistent with other studies, we observed that 2 μg of synthetic melanin (Sigma) was sufficient to inhibit PCR [5, 8]. BSA is widely used in PCR methods, as it relieves interference caused by several substances, including humic acids [14], bone [15], blood [16], meat [16], feces [16], and heme-containing compounds [17]. Here, 40 ng/μl BSA greatly relieved the PCR-inhibitory effects of the synthetic melanin in the DNA template extracted from cultured cells. This result differed slightly from Giambernardi reports, where 600 ng/μl BSA was required to ease the inhibitory effects of melanin on DNA amplification [8]. This difference is likely a result of the different cell types that were used in this study. However, the effects of BSA on relieving PCR inhibition appear to be concentration-limited because additional BSA did not overcome inhibition when the melanin concentration was increased. Interestingly, we observed that when BSA was added to pigmented DNA samples, 40/50 (80%) samples were correctly amplified with acceptable intra-assay reproducibility and established a conclusive *BRAF* mutation result. Additionally, *BRAF* mutations could be easily observed on sequencing electropherograms in these cases, demonstrating the usefulness of this pre-treatment procedure.

An alternative strategy to overcome PCR inhibition is to dilute the DNA template for amplification. This strategy has been shown to improve PCR performance [18, 19]. In cell lines, a minimum dilution of 1/10 was required to reduce the melanin concentration while still obtaining significant *BRAF* amplification. This resulted in the amplification of 2 ng of template DNA. However, this strategy was often undesirable—and indeed sometimes impossible—when analyzing our clinical melanoma cohort. Dilution rendered the DNA concentration below the minimum template amounts required to yield full results, probably due to elevated concentrations of a remaining PCR inhibitor or a low amount of DNA template.

Finally, an additional way to remove PCR inhibitors is through the use of purification methods. Here, we selected the NucleoSpin^®^ Kit for the melanin cleanup procedure. Running DNA extracts derived from cell lines and clinical melanoma samples through this column generally provided efficient DNA amplification. However, the detection of *BRAF* mutations was effective in only 48% of cases due to the lack of interpretable HRM profiles. Although this kit has proven efficient in removing hematin, indigo, and urea, it may not remove DNA-bound PCR inhibitors such as melanin [7]. However, other kits or procedures may remove PCR inhibitors with greater efficiency. Co-purified melanin pigments was largely eliminated from RNA samples after passing through polyacrylamide-based beads (Bio-Gel P-60) [20]. More recently, GENECLEAN^®^ II purification kit (Vista) has been shown to increase the percentage of cases successfully analysed from 27.3% to 95.4% for Sanger sequencing, and from 95.4% to 100% for a new Cast-PCR approach [21]. Lastly, and most interestingly, we observed that the addition of BSA to the pre-PCR clean-up method allowed the detection of a V600K *BRAF* mutation that was initially missed when the procedure was used individually.

Based on these considerations, our recommendations to maintain, and perhaps enhance, the screening capabilities of HRM analysis for samples extracted from paraffin-embedded tissue or mutation identification with Sanger sequencing would include the use of i) a pre-PCR method in weakly or moderately pigmented melanoma DNA samples and ii) a double pre-PCR procedure optimization in highly pigmented DNA samples.

In conclusion, this study demonstrates that the PCR-inhibitory effects of biological samples can be reduced or eliminated by the use of an appropriate procedure. Although dilution methods or chromatographic columns could overcome inhibition in some cases, additional PCR facilitators such as BSA should be considered prior to PCR runs. However, if DNA amplification cannot be improved this way, further purification or alternative extraction methods may be beneficial.

## Supporting Information

S1 Fig Representative melting curves obtained by HRM analysis of tumor samples. (A) Normalized melting curves. (B) Difference graph derived from the normalized data. The purple curve corresponds to a BRAF-wildtype sample, the pink curve to an inconclusive sample, the green curve to a *BRAF* V600E mutant sample, and the blue curve to a *BRAF* V600K mutant sample.
(TIF)
