# Supplementary figures and images for: Comparative Methods to Improve the Detection of BRAF V600 Mutations in Highly Pigmented Melanoma Specimens
Source: PLoS One. 2016 Jul 28;11(7):e0158698. doi: 10.1371/journal.pone.0158698 (PMC4965116; doi:10.1371/journal.pone.0158698)

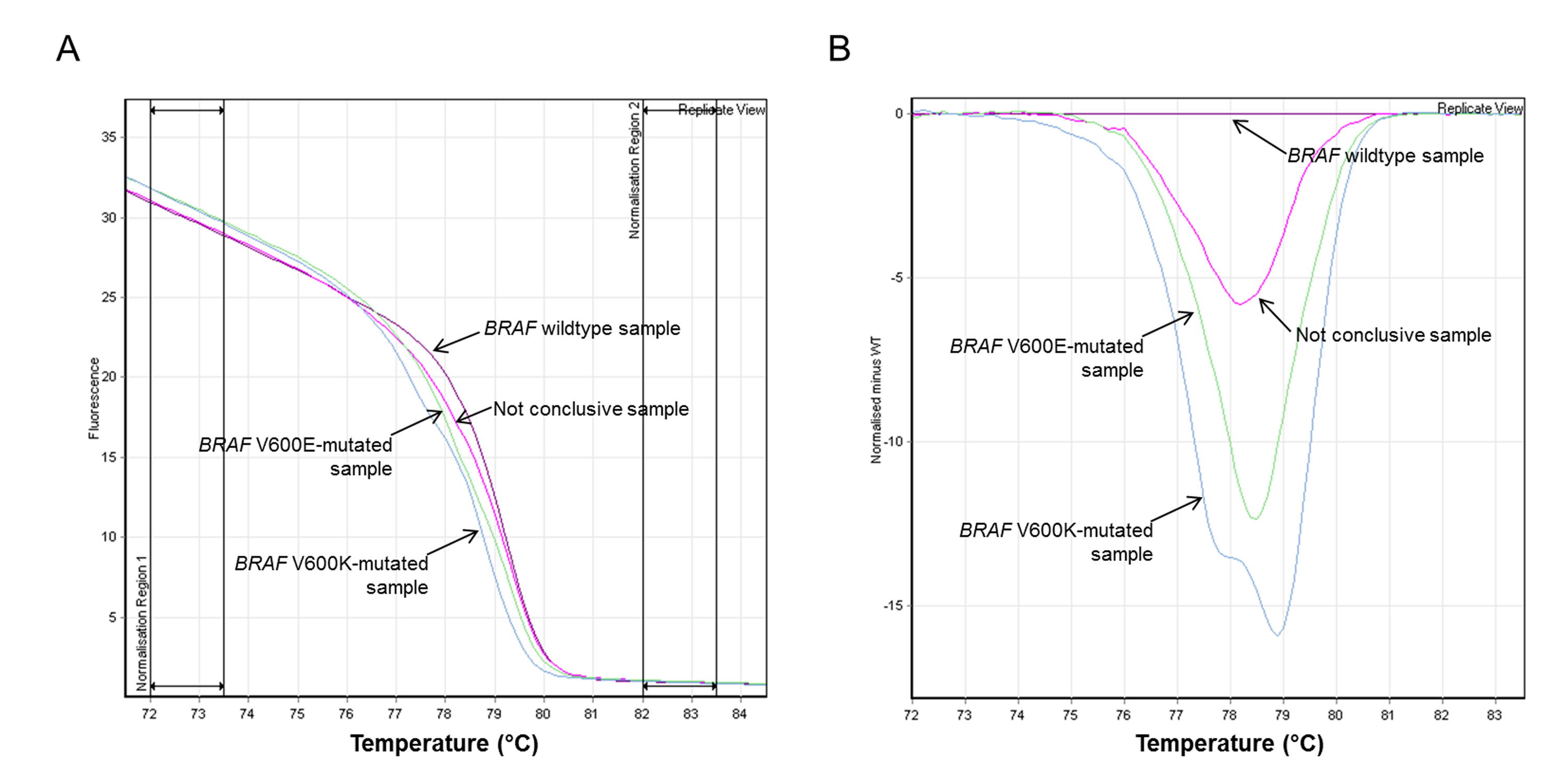

Supplement: S1 Fig — (A) Normalized melting curves. (B) Difference graph derived from the normalized data. The purple curve corresponds to a BRAF-wildtype sample, the pink curve to an inconclusive sample, the green curve to a BRAF V600E mutant sample, and the blue curve to a BRAF V600K mutant sample. (TIF) [file pone.0158698.s001.tif]
